# Characteristics of Traveler with Middle East Respiratory Syndrome, China, 2015

**DOI:** 10.3201/eid2112.151232

**Published:** 2015-12

**Authors:** Wen Da Guan, Chris Ka Pun Mok, Zi Lin Chen, Li Qiang Feng, Zheng Tu Li, Ji Cheng Huang, Chang Wen Ke, Xilong Deng, Yun Ling, Shi Guan Wu, Xue Feng Niu, Ranawaka A Perera, Yuan Da Xu, Jincun Zhao, Lin Qi Zhang, Yi Min Li, Rong Chang Chen, Malik Peiris, Ling Chen, Nan Shan Zhong

**Affiliations:** State Key Laboratory of Respiratory Disease, First Affiliated Hospital of Guangzhou Medical University, Guangzhou, China (W.D. Guan, Z.T. Li, S.G. Wu, X.F. Niu, Y.D. Xu, J. Zhao, Y.M. Li, R.C. Chen, L. Chen, N.S. Zhong);; The University of Hong Kong, Hong Kong, China (C.K.P. Mok, R.A. Perera, M. Peiris);; Huizhou Municipal Central Hospital, Huizhou, China (Z.L. Chen, Y. Ling);; Guanzhou Institute of Biomedicine and Health, Guangzhou (L.Q. Feng, L. Chen);; Guangdong Inspection and Quarantine Technology Center, Guangzhou (J.C. Huang);; Guangdong Center for Disease Control and Prevention, Guangzhou (C.W. Ke);; Guangzhou Eighth People‘s Hospital, Guangzhou (X. Deng);; Tsinghua University School of Medicine, Beijing, China (L.Q. Zhang)

**Keywords:** Middle East respiratory syndrome, MERS, Middle East respiratory syndrome coronavirus, MERS-CoV, coronavirus, viruses, cytokines, outbreak, South Korea, China

**To the Editor:** A traveler returning from the Middle East initiated an outbreak of Middle East respiratory syndrome (MERS) in South Korea in 2015, which resulted in 186 cases and 36 deaths (*1*–*3*). We report a case of MERS in a 43-year-old man from South Korea who acquired this disease during this outbreak (Technical Appendix Figure 1) (*4*).

The National Health and Family Planning Commission of China determined that collection of data for this patient was part of a public health investigation of an emerging outbreak. Therefore, informed consent was not required. This study was approved by the ethical committee of the First Affiliated Hospital of Guangzhou Medical University.

The patient had been receiving thiamazole for 7 years for hyperthyroidism. He had contact with the index case-patient during the outbreak in South Korea on May 16, 2015. On May 25, the patient traveled to Hong Kong and then to Huizhou, China. He was hospitalized in China on May 28 (day 7 of illness). At admission, he had a high fever (temperature 39.5°C) and a dry cough. Chest radiography on day 7 showed mild bilateral ground glass opacities in the lower lung (Technical Appendix Figure 1, panel B).

The patient was given oseltamivir (150 mg, 2×/day for 2 days) until identified as being infected with Middle East respiratory syndrome coronavirus (MERS-CoV) on day 8 by real-time reverse transcription PCR. He was given ribavirin (2.0 mg on day 8; 0.6 mg 3×/d on days 9–16; and 0.6 mg 2×/d on days 17–19) and 135 μg of peginterferon αa-2a by intravenous injection on day 8 (Technical Appendix Table 2). Thrombocytopenia and a decrease in the hemoglobin level developed, which might have been related to use of ribavirin (Technical Appendix Table 1).

Chest radiography on June 1 (day 11) showed increased bilateral consolidation of the patient’s lower lung (Technical Appendix Figure 1, panel C). He was given intravenous immunoglobulin, antimicrobial drugs, and thymosin α1. His body temperature returned to normal on day 14 (Technical Appendix Figure 2). Chest radiography on day 35 showed resolution of bilateral lung infiltrations (Technical Appendix Figure 1, panel D). He was discharged on day 36.

Viral RNA was detected in sputum and fecal specimens up to day 26 of illness. Virus load (*5*) in sputum specimens collected on days 11–15 were lower than in specimens obtained on days 16–18 (Technical Appendix Figure 3, panel A). Swab samples collected on days 13 and 15 from the patient’s palm, mobile telephone, blanket, and bed railings, and from his hospital room floor were negative for viral RNA.

Concentrations of proinflammatory cytokines and chemokines (interferon-α, interferon-inducible protein 10, monocyte chemoattractant protein-1, interleukin 6 [IL-6], IL-10, tumor necrosis factor-α, IL-8, macrophage inflammatory protein-α [MIP-1α], MIP-1β, and IL-1β) were determined for serial serum samples. Interferon-α, interferon-inducible protein 10, monokine induced by interferon-γ, IL-6, monocyte chemoattractant protein-1, and IL-8 were detected on day 11 of illness but levels decreased as the patient clinically improved (Technical Appendix Figure 3, panel B).

The peginterferon αA2 the patient was given on day 8 might have influenced his plasma interferon-α levels (*6*). However, a previous study also showed increased levels of interferon-α in a patient who survived MERS-CoV infection but not in a person who died of MERS (*7*). Although MERS-CoV evades induction of innate immune responses by cell types, the virus elicits interferon responses in plasmacytoid dendritic cells in vitro (*8*). Levels of tumor necrosis factor-α, MIP-1α, MIP-1β, IL-10, and IL-1β did not increase in any of these specimens.

Peripheral blood mononuclear cells (PBMCs) obtained on day 24 of illness showed a strong specific T-cell response against MERS-CoV spike protein but not against severe acute respiratory syndrome coronavirus (SARS-CoV) spike protein (Technical Appendix Figure 3, panel C). PBMCs from persons who were infected with SARS-CoV in 2003, as well as healthy persons, showed low-level T-cell responses against MERS-CoV spike protein, although some persons with a history of SARS still had detectable responses to SARS-CoV spike protein. It was reported that T-cell responses to SARS-CoV were directed against spike and nucleocapsid proteins (*9*). We did not have sufficient PBMCs to test T-cell responses against nucleocapsid protein.

Results for MERS-CoV antibody were negative at day 11 of illness by MERS-CoV spike pseudotype assay (MERS-S ppNT), microneutralization, 50% plaque reduction neutralization test (PRNT_50_), and S1 ELISA (EUROIMMUN AG, Lübeck, Germany). The patient showed seroconversion by day 14. MERS-S ppNT and PRNT_50_ provided earlier evidence of seroconversion (day 15) and higher antibody titers than the microneutralization, (day 18) (Technical Appendix Figure 3, panel D). Potent T-cell responses were elicited to MERS-CoV spike protein. These responses did not show cross-reactivity with SARS-CoV spike protein.

The MERS-S ppNT, which does not require Biosafety Level 3 containment, had sensitivity equivalent with that of PRNT_50_, which requires containment. Thus, MERS-S ppNT is a sensitive and specific assay for detecting neutralizing antibody against MERS-CoV. The sensitivity and specificity of this assay have been well-documented with serum samples from dromedary camels and other animals (*10*).

**Technical Appendix.** Methods used for testing and results for a traveler with Middle East respiratory syndrome, China, 2015.
