# Supplementary material for: Characteristics of Traveler with Middle East Respiratory Syndrome, China, 2015
Source: Emerg Infect Dis. 2015 Dec;21(12):2278–80. doi: 10.3201/eid2112.151232 (PMC4672405; doi:10.3201/eid2112.151232)
Supplement: Supplementary file 1 — Technical Appendix. Methods used for testing and results for a traveler with Middle East respiratory syndrome, China, 2015. [file 15-1232-Techapp-s1.pdf]

Article DOI: http://dx.doi.org/10.3201/eid2112.151232

Characteristics of Traveler with Middle East Respiratory Syndrome, China, 2015

Technical Appendix

Methods

Detection of Virus in Samples

Throat swab, nasal swab, sputum, urine, serum, fecal, and environmental samples were collected from the patient and placed in viral transport medium. Viral RNA was extracted from samples by using the QIAamp MinElute Virus Spin Kit (QIAGEN, Valencia, CA, USA) according to the manufacturer's instructions. Middle East respiratory syndrome coronavirus (MERS-CoV) was detected by using real-time reverse transcription PCR with primers upE-Fwd (5′-GCAACGCGCGATTCAGTT-3′) and upE-Rev (5′-GCCTCTACACGGGACCCATA-3′), and probe upE-Prb (6-carboxyfluorescein[FAM]-5′-CTCTTCACATAATCGCCCCGAGCTCG-3′-6-carboxy-N,N,N,N′-tetramethylrhodamine [TAMRA]).

Determination of Cytokine Levels

Levels of interleukin 8 (IL-8), interferon-inducible protein 10, interferon-α, macrophage inflammatory protein-α, macrophage inflammatory protein-β, monocyte chemoattractant protein-1, monokine induced by interferon-γ (IFN-γ), IL-1β, tumor necrosis factor-α, IL-6, and IL-10 in serum samples were determined by using a Cytometric Bead Assay Human Inflammatory Cytokine Kit (BD Biosciences, San Jose, CA, USA) according to the manufacturer's instructions. Data acquisition was performed by using a BD LSR Fortessa Analyzer (BD Biosciences). Data were analyzed by using cytometric bead assay analysis software (BD Biosciences).

IFN-γ ELISPOT Assay

Whole blood samples from healthy donors, health care workers who had been infected with SARS-CoV, and the MERS patient were collected into anticoagulant tubes containing EDTA, and the peripheral blood mononuclear cells (PBMCs) were

isolated by using standard density gradient centrifugation. An IFN-γ ELISPOT assay was performed as follows. A 96-well plate containing Immobilon-P membrane (MSIPS4510; Millipore, Billerica, MA, USA) was incubated with monoclonal anti-monkey IFN-γ (CT610–10; U-Cytech, Utrecht, the Netherlands) at 4°C overnight and blocked with RPMI medium 1640 (no. 11875–093; Gibco, Grand Island, NY, USA) containing 0.05 mmol/L 2-mercaptoethanol, 1 mmol/L sodium pyruvate, 2 mmol/L L-glutamine, 10 mmol/L HEPES, and 10% fetal bovine serum (no. SH30070; HyClone, Logan, UT, USA) at 37°C for 2 h.

PBMCs were plated at a density of 2.5×10^5 cells/well. Recombinant MERS-CoV spike protein (no. 40069-V08B; Sino Biologic Inc., North Wales, PA, USA) and SARS-CoV spike S1 subunit (no. 40150-V08B1; Sino Biologic Inc.) were added to the wells at a final concentration of 10 μg/mL to stimulate PBMCs. Phosphate-buffered saline was used for mock stimulation. At 24-h poststimulation, IFN-γ was detected by using biotinylated anti-monkey IFN-γ (CT610–10; U-Cytech), and color was developed by using nitro-blue tetrazolium and 5-bromo-4-chloro-3'-indolyphosphate (Pierce, Rockford, IL, USA). Spots were counted by using an ELISPOT reader (Bioreader 4000; BIOSYS, Germany), and the results were reported as number of spot-forming cells/million PBMCs.

Microneutralization Test

Serum samples were heat-inactivated (56°C for 30 min) and mixed with equal volumes of two hundred 50% tissue cultures infectious doses of MERS-CoV. After 1 h of incubation at 37°C, 35 μL of virus/serum mixture was added in quadruplicate to Vero cell monolayers in 96-well microtiter plates. After 1 h of adsorption, the virus/serum mixture was removed, 150 μL of culture medium was added to each well, and plates were incubated for 3 days at 37°C in an atmosphere of 5% CO_2 in a humidified incubator. Cytopathic effect was read at day 3 postinfection. The highest serum dilution that completely protected the cells from a cytopathic effect in half of the wells was taken as the neutralizing antibody titer and was estimated by using the Reed-Muench method (*1*).

MERS-CoV Spike Pseudoparticle Neutralization Assay

MERS CoV pseudoparticles were incubated for 1 h with an equal volume of serum dilution at 4°C. After incubation for 1 h, serum/virus mixture was added to Vero cell monolayers in triplicate in a 96-well format. At day 3 posttransduction,

luciferase activity was measured by using a Microbeta Luminometer (PerkinElmer, Waltham, MA, USA). The highest serum dilution giving 90% reduction of luciferase activity was used as the antibody titer (*2*).

MERS CoV S1 ELISA

A commercially available (EUROIMMUN AG, Lübeck, Germany) MERS CoV S1 ELISA was used. In brief, serum dilutions, a calibrator, and controls were added to MERS CoV–coated plates and incubated for 30 min. Plates were washed 3 times and peroxidase labeled anti-human IgG was added and incubated for 30 min. Substrate solution was added and incubated for 15 min at room temperature. Reactions were stopped, and absorbance was measured at 450 nm using a reference wave length of 650 nm. The ratio between the extinction values of control/sample and calibrator was calculated. A ratio >0.8 was considered a positive result (*3*).

Plaque Reduction Neutralization Test

Equal volumes of each serum dilution and 40–60 PFU of MERS CoV/24-well plate were mixed and incubated for 1 h at 37°C in an atmosphere of 5% CO_2 in a humidified incubator. After incubation, virus/serum mixture was added to Vero cell monolayers in a 24-well format in duplicate. Plates were incubated for 1 h to enable absorption before overlaying with agarose. After 3 days, cells were fixed with 10% paraformaldehyde and stained. Titers are expressed as serum dilutions resulting in plaque inhibition $\geq$90%.

References

<jrn>1. Reed LJ, Muench H. A simple method of estimating fifty percent endpoints. American Journal of Hygiene. 1939;27:493–7.</jrn>

<jrn>2. Hemida MG, Perera RA, Al Jassim RA, Kayali G, Siu LY, Wang P, et al. Seroepidemiology of Middle East respiratory syndrome (MERS) coronavirus in Saudi Arabia (1993) and Australia (2014) and characterisation of assay specificity. Euro Surveill. 2014;19:pii: 20828. PubMed</jrn>

<jrn>3. Müller MA, Meyer B, Corman VM, Al-Masri M, Turkestani A, Ritz D, et al. Presence of Middle East respiratory syndrome coronavirus antibodies in Saudi Arabia: a nationwide, cross-sectional, serological study. Lancet Infect Dis. 2015;15:559–64. PubMed http://dx.doi.org/10.1016/S1473-3099(15)70090-3</jrn>

Technical Appendix Table 1. Biochemical parameters for a 43-year-old traveler with Middle East respiratory syndrome, China, 2015*

Parameter	Reference range	Days after illness onset 9	11	16	21	23	29	33
Total leukocytes, × 10^9 cells/L	4.0–10.0	3.22	3.06	3.6	4.9	4.8	4.6	7.3
Neutrophils, × 10^9 cells/L	1.8–6.3	2.35	1.71	2.0	2.6	2.5	2.4	4.6
Lymphocytes, × 10^9 cells/L	1.1–3.2	0.65	1.02	1.0	1.7	1.7	1.7	2.0
Platelets, × 10^9/L	125–350	81	113	253	378	362	274	263
Hemoglobin, g/dL	130–175	142	149	117	119	119	109	106
Aspartate aminotransferase, U/L	17–59	24	26	–	35	34	35	44
Creatinine, μmol/L	71–133	54	65	–	52	51	54	59
Creatine kinase, U/L	55–170	–	–	–	21	41	29	41
Creatine kinase isoenzyme, U/L	0–25	8	4	–	6	6	6	4

*–, data not collected.

Technical Appendix Table 2. Clinical characteristics for a 43-year-old traveler with Middle East respiratory syndrome, China, 2015*

Characteristic	Result or value
Clinical features	
Fever	Yes
Highest temperature, °C	39.5
Cough	No
Sputum production	Yes
Shortness of breath	Yes
Chills	No
Complications	
Pneumonia	Yes
Acute respiratory distress syndrome	Yes
Shock	No
Bacterial co-infection	No
Treatment	
Oxygen therapy	Yes, inhalation oxygen with nasal catheter
Mechanical ventilation	No
Antivirus	Oseltamivir (150 mg, 2×/d on days 7 and 8); ribavirin (2.0 mg/d on day 8; 0.6 mg 3×/d on days 8–16; 0.6 mg 2×/d on days 17–19); peginterferon-α 2a (135 μg/d iv on day 8)
Antimicrobial drug	
Ceftriaxone sodium	2.0 g/d iv on days 10–13
Meropenem	2.0 g 3×/d iv on days 13–26
Glucocorticoids	No
Immune regulation therapy	
Immune globulin	20 g/d iv on days 11–17
Thymosin alpha-1	1.6 mg/d iv on days 14–18; 1.6 mg 2×/d iv on days 19–26
Clinical outcome	Discharged

*iv, intravenous.

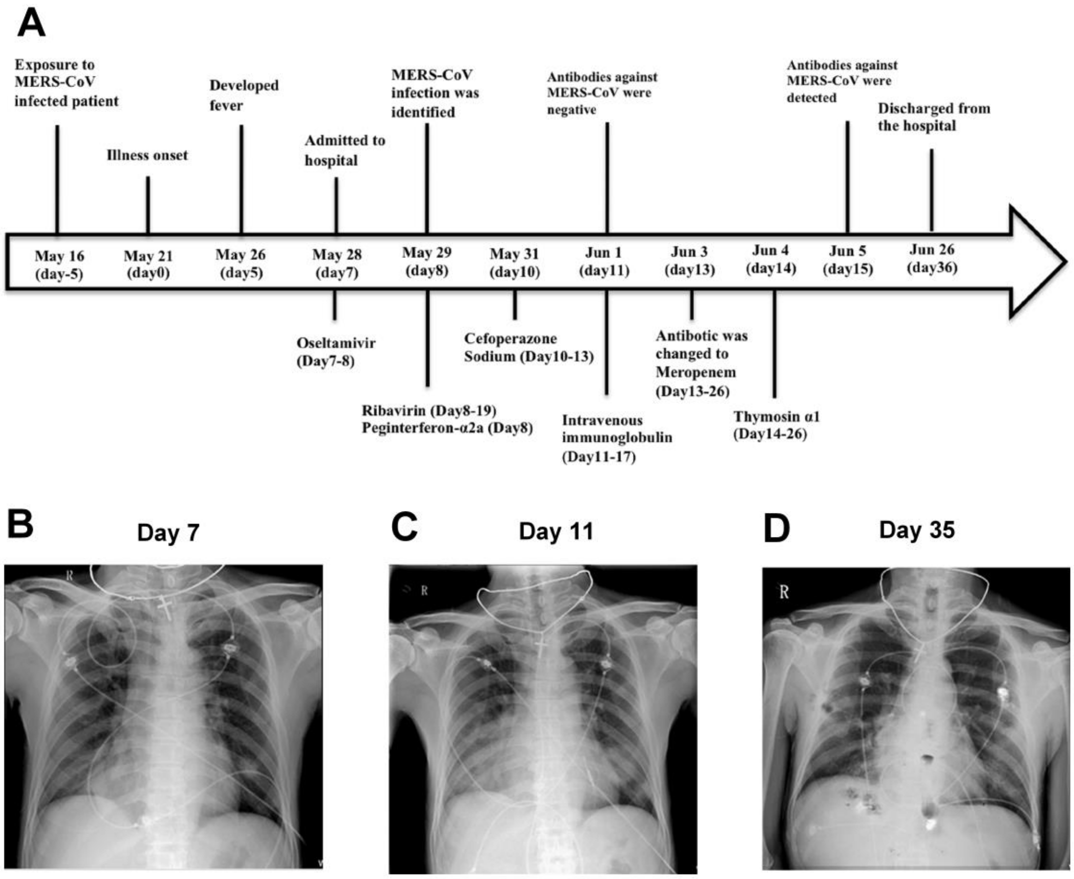

Technical Appendix Figure 1. A) Timeline of clinical course of a 43-year-old traveler with Middle East respiratory syndrome, China, 2015. MERS-CoV, Middle East respiratory syndrome coronavirus. B–D) Chest radiographs on days 7, 11, and 35.

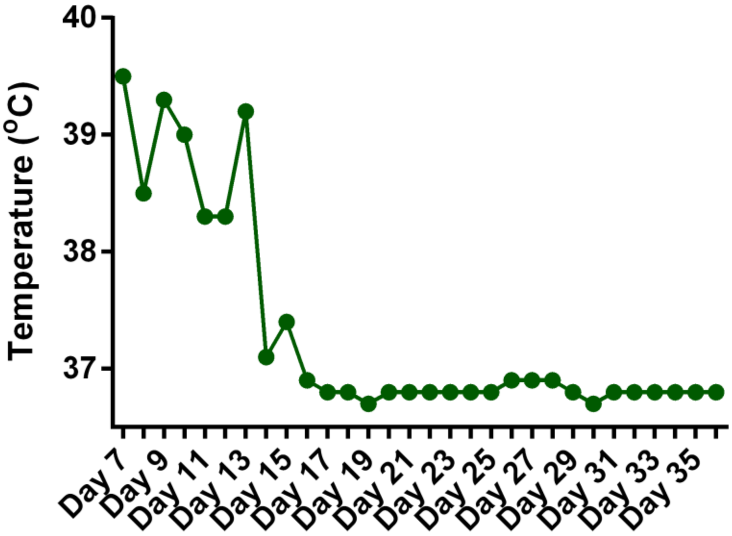

Technical Appendix Figure 2. Body temperature of 43-year-old traveler with Middle East respiratory syndrome, by day of hospitalization, China, 2015.

A

The viral load detected from varies collection of samples during hospitalization

	MERS-CoV RNA copy numbers (log10)												
Day of onset	11	12	13	14	15	16	17	18	19	22	25	26	36
Sputum on demand	3.0	3.9	3.6	2.0	2.4	N.C.	N.C.	N.C.	2.8	2.2	N.C.	N.C.	-
Expectorated sputum*	N.C.	N.C.	N.C.	N.C.	N.C.	6.0	6.1	2.5	N.C.	N.C.	2.0	3.0	N.C.
Throat Swab	-	-	-	-	-	-	-	N.C.	N.C.	N.C.	N.C.	N.C.	N.C.
Nasal Swab	-	-	N.C.	-	-	N.C.	N.C.	N.C.	N.C.	N.C.	N.C.	N.C.	N.C.
Serum	-	N.C.	N.C.	-	-	-	N.C.	-	N.C.	-	N.C.	-	N.C.
Stool	N.C.	N.C.	N.C.	2.5	N.C.	N.C.	N.C.	N.C.	3.5	N.C.	-	2.1	N.C.
Urine	N.C.	N.C.	N.C.	N.C.	N.C.	N.C.	N.C.	-	-	N.C.	-	N.C.	N.C.
Environmental samples	N.C.	N.C.	-	N.C.	-	N.C.	N.C.	N.C.	N.C.	N.C.	N.C.	N.C.	N.C.

Samples with threshold cycle lower than 40 were regarded as negative; N.C. : Not collected; - : Negative detection; Environmental samples : Palm, Pass Box, Bed railings(Day 13); Palm, Bed railings, Blanket, mobile phone, Floor (Day 15); *: A sputum cup was provided for the patient to collect sputum when he coughed. The sputum cup was collected in the morning for analysis.

B

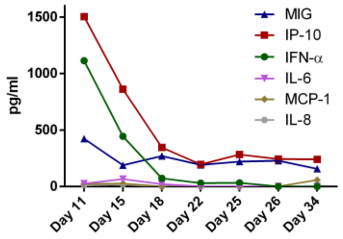

C

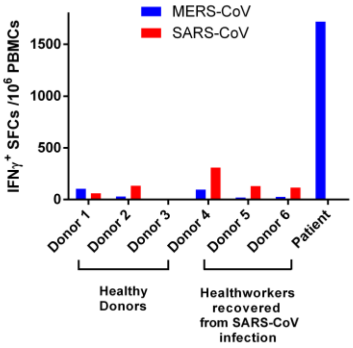

D

Detection of antibodies against MERS-CoV by using different assays

	MERS-CoV antibody				
Days after onset of illness	ppNT titer+	Micro-neutralization titer	PRNT# (90% reduction)	PRNT (50% reduction)	S1-ELISA* (Calibrator/sample extinction ratio)
11	<1:10	<1:10	<1:10	<1:10	0.03 (Negative)
15	1:80	1:10	1:40	1:80	1.32 (Positive)
18	1:320	1:40	1:80	1:160	2.25 (Positive)
22	1:320	1:40	1:80	1:160	2.53 (Positive)
25	1:320	1:40	-	-	2.46 (Positive)
26	1:320	1:40	1:160	1:320	2.81 (Positive)
34	1:640	1:80	1:160	1:320	3.1 (Positive)

*ELISA optical density cut-off for positive result = 0.8; + ppNT: MERS-CoV spike pseudotype assay;
#PRNT: Plaque reduction neutralization titer.

Technical Appendix Figure 3. Clinical parameters for a 43-year-old traveler with Middle East respiratory syndrome, China, 2015. A) Virus load. MERS-CoV, Middle East respiratory syndrome coronavirus. B) Cytokine levels and antibody titers. MIG, monokine induced by interferon-γ; IP10, interferon-inducible protein 10; IFN-α, interferon-α; IL-6, interleukin 6; MCP-1, monocyte chemoattractant protein-1. C) T-cell response of peripheral blood mononuclear cells (PBMCs) after challenge with MERS-CoV spike protein. SARS-CoV, severe acute respiratory syndrome coronavirus; IFNγ, interferon-γ; SFCs, spot-forming cells. D) Detection of antibodies against MERS-CoV by different assays.
